# Effect of tamoxifen with or without gonadotropin-releasing hormone analog on DXA values in women with breast cancer

**DOI:** 10.1038/s41598-021-82824-x

**Published:** 2021-02-09

**Authors:** Eun Heui Kim, Yun Kyung Jeon, Kyoungjune Pak, Taewoo Kang, Kyung-Eun Kim, Seong-Jang Kim, In-Joo Kim, Keunyoung Kim

**Affiliations:** 1 Division of Endocrinology and Metabolism, Department of Internal Medicine and Biomedical Research Institute, Pusan National University Hospital, Busan, Republic of Korea; 2 Department of Nuclear Medicine and Biomedical Research Institute, Pusan National University Hospital and School of Medicine, Pusan National University, Busan, Republic of Korea; 3 Busan Cancer Center (Breast Cancer Clinic) and Biomedical Research Institute, Pusan National University Hospital, Busan, Republic of Korea; 4 Department of Nuclear Medicine and Research Institute for Convergence of Biomedical Science and Technology, Yangsan Pusan National University Hospital, Yangsan, Republic of Korea

**Keywords:** Endocrinology, Metabolic bone disease

## Abstract

The purpose of this study was to compare the changes in DXA values including trabecular bone score (TBS) and bone mineral density (BMD) of lumbar spine (LS) and femur according to the hormone therapies including tamoxifen (TMXF) treatment with or without gonadotropin releasing hormone analog (GnRH analog) in women with breast cancer. We enrolled 119 women with breast cancer who had undergone breast-conserving surgery or mastectomy followed by TMXF treatment for postmenopausal women (TMXF group, n = 63, 52.9%) or by combination therapy of TMXF combined with GnRH analog for premenopausal women (TMXF + GnRH group, n = 56, 47.1%) from December 2013 to December 2017. The median follow-up period was 13 months (interquartile range [IQR], 12.0–14.75) for TMXF group and 13.5 months (IQR, 12.00–16.00) for TMXF + GnRH group, respectively. Patients did not receive bone-modifying therapy. The baseline dual-energy X-ray absorptiometry (DXA) scan before breast cancer surgery and follow-up DXA during hormone therapy. Comparing the first and follow-up DXA results, BMD in LS were significantly decreased in both TMXF (*P* < 0.001, mean difference: − 0.06) and TMXF + GnRH (*P* < 0.001, mean difference: − 0.09) groups. BMD values of femoral neck (*P* = 0.0011, mean difference: − 0.01) and total femur (*P* < 0.001, mean difference: − 0.03) was significantly changed between the baseline and follow-up DXA in TMXF + RnRH group. In the TMX group, a significant changed occurred in the BMD in total femur (*P* < 0.001, mean difference: − 0.030) but not the BMD of femoral neck (*P* = 0.095, mean difference: − 0.007). Regarding TBS, no significant change was found in the TMXF (*P* = 0.574, mean difference: − 0.004) group, whereas there was a significant decrease in TBS in the TMXF + GnRH (*P* < 0.001, mean difference: − 0.02) group during follow-up. TBS is more sensitive in reflecting the bone microarchitecture changes by TMXF or GnRH agonist in breast cancer patients than BMD. This finding demonstrates that TBS can be a useful parameter to detect bone microarchitectural changes in clinical applications.

## Introduction

Hormone-receptor positive cancers are the most common type of breast cancer, accounting for 75% of all breast cancers^1–3^. The recommended treatment is adjuvant endocrine therapy with aromatase inhibitors or tamoxifen (TMXF), a selective estrogen receptor modulator. TMXF is less effective than aromatase inhibitors in hormone-receptor positive breast cancer^4–7^; however, it is used for patients with low risk or those who cannot tolerate aromatase inhibitor.

In premenopausal women, it has been reported that TMXF with ovarian function suppression moderately improved disease-free survival compared to TMXF alone^8^. From the perspective of bone health, ovarian function suppression using gonadotropin-releasing hormone analog (GnRH analog) has the potential to increase the osteoporotic fracture risk of patients because of its estrogen-depriving effect^9–11^. It has been reported that TMXF could increase bone mineral density (BMD) because of its partial estrogen-receptor agonistic effects on the skeleton^2,3,12^. However, a recent study reported that TMXF did not have a preventive effect on the risk of fracture in postmenopausal women, and increased the risk of fracture in premenopausal women^13^. In premenopausal women, TMXF competes with estradiol for estrogen-receptor binding sites as a partial antagonist, leading to net bone loss^13^.

Qualitative parameters of bone, such as trabecular bone score (TBS), could predict osteoporotic fractures independent of BMD acquired from dual-energy X-ray absorptiometry (DXA)^14^. In a breast cancer prevention study, aromatase inhibitor therapy for 2 years compared to placebo was associated with loss of volumetric BMD and cortical thickness at a radius of − 4.3% and − 6.8%, respectively, whereas bone loss by DXA was < 2% at all sites^15^. In a study about the effect of endocrine treatment on densitometric results, results were discordant between the change in BMD and TBS in a TMXF group and an aromatase inhibitor group of postmenopausal women with breast cancer^16^.

Studies regarding the effect of combination therapy with TMXF and GNRH analog on bone changes are lacking. We used BMD and TBS to investigate how these endocrine treatments affect bone microarchitecture in breast cancer patients.

Therefore, we compared the changes in both TBS and BMD to evaluate the effect of TMXF treatment in postmenopausal women or combination therapy with TMXF and GnRH analog in premenopausal women with breast cancer.

## Materials and methods

### Patients

The study is a retrospective cross-sectional study. We recruited 119 women with breast cancer who received breast-conserving surgery or mastectomy for the treatment of breast cancer, followed by adjuvant endocrine therapy either by monotherapy of TMXF or combination therapy of TMXF and GNRH analogs between December 2013 and December 2017. All patients were followed-up for a median period of 13.00 months (interquartile range [IQR], 12.00–15.00). The median follow-up period was 13.00 months (IQR, 12.00–14.75) for TMXF group and 13.50 months (IQR, 12.00–16.00) ) for TMXF + GnRH group, respectively. Patients were eligible for the study if they met the following criteria: patients being treated with TMXF only for postmenopausal women (TMXF group), or with TMXF combined with GnRH for premenopausal women (TMXF + GnRH group), for more than 6 months, and who had no bone modifying therapy at any time. Prior chemotherapy was not considered for exclusion because the proportion of the patients was not different between postmenopausal and premenopausal status.

This study was approved by the Institutional Review Board of Pusan National University Hospital, which waived the requirement for written consent (IRB No. 1801-003-062). All methods were performed in accordance with relevant guidelines and regulations.

### DXA

Both sites of lumbar spine (LS) and femur were evaluated using DXA scan images (Lunar Prodigy; GE Medical Systems, Milwaukee, WI, USA) and analyzed using Encore software (ver. 13.0; GE Healthcare, Madison, WI, USA). Baseline DXA scans were performed prior to breast cancer surgery, and follow-up studies were performed during endocrine therapy. We adopted the baseline DXA and the first follow-up DXA images for the analysis. The BMD (g/cm^2^) was obtained in LS, femoral neck (FN) and total femur (TF). TBS of the LS was derived from average values of L1–L4. A daily calibration and quality assurance test were performed, and the coefficient of variation for precise measurement of the BMD of the LS was 0.34%. The least significant change (LSC) at 95% confidence level were within 0.003 g/cm^2^ for BMD of LS, 0.048 g/cm^2^ for BMD of FN, 0.033 g/cm^2^ for BMD of TF and within 0.053 for TBS. All patients were evaluated with T-scores for the diagnosis, because at the time of the acquisition of DXA, all patients were already in perimenopausal or menopausal status due to physiologic causes or GnRH therapy. The T-score was defined according to the number of standard deviations (SDs) from the mean BMD of a reference group from the general population and matched for sex at 25–35 years of age. T-scores ≤  − 2.5 SDs from the reference mean were defined as osteoporosis; T-scores between  − 2.5 and  − 1.0 SDs from the reference mean were defined as osteopenia; T-scores ≥  − 1.0 SD from the reference mean were defined as normal. The TBS of the LS was extracted from the DXA file using iNsight software (ver. 3.0.2.0; Medimaps, Pessac, France). We used the following TBS cutoffs proposed by an international working group of TBS users for postmenopausal women: normal for TBS values ≥ 1.35, degraded microarchitecture for values between 1.20 and 1.35, and more degraded for values ≤ 1.20^17^.

### Statistical analyses

Values of all non-normally distributed variables are expressed as medians and interquartile ranges (IQR; 25–75%). A Mann–Whitney U-test was used to compare continuous variables for the two groups. For comparing the categorical data of the groups, the chi-squared test was used. A paired t-test was used to compare the average differences in values between the baseline DXA and the follow-up DXA. The statistical analyses were performed using the MedCalc software (version 16.4.3; MedCalc, Mariakerke, Belgium) and RStudio (version 1.2b; RStudio, MA, USA). A *P* value less than 0.05 was regarded as indicative of significance.

## Results

### Baseline characteristics

A total of 119 women were enrolled; 63 (52.9%) were included in the TMXF group and 56 (47.1%) were included in the TMXF + GnRH group. Tamoplex was used as TMXF therapy with the dose of 20 mg once a day or 10 mg twice a day. Zoladex was added as a GnRH therapy for all patients in TMXF + GnRH group. For GnRH therapy, 3.6 mg of Zoladex was administered subcutaneously every 28 days. The average duration of use of TMXF was 57.5 months with interquartile range [IQR] of 51–59 months. The median duration of use of GnRH was 40.50 months with IQR of 26–54 months. The age (median age: 48 years; IQR, 44–77] for TMXF group versus 40 years; IQR, 30–49] for TMXF + GnRH group, *P* < 0.001) was significantly different between the two groups. Other characteristics including BMI, height, weight, stage of the breast cancer, hormonal status, type of operation and chemotherapy were not significantly different between two groups. The proportion of TMXF group who received radiotherapy was higher than that of TMXF + GnRH group (82.5% vs. 57.1%, *P* = 0.003). The median follow-up period was 14 months (IQR, 12.0–15.0) for all patients and there was no significant difference between the two groups (median: 13.00 months [IQR, 12.00–14.75) versus 13.50 months [12.00–16.00], *P* = 0.246). Demographic information and baseline characteristics are shown in Table 1.

**Table 1:** Comparative baseline demographic and clinical characteristics.

Characteristics	TMXF	TMXF + GnRH	*P* value
No. of patients	63	56	
Age (years)	48 (44–77)	40 (30–49)	< 0.001
BMI (kg/m^2^)	23.10 (21.08–25.10)	21.80 (19.75–23.95)	0.140
Height (cm)	158.30 (153.63–160.90)	159.55 (156.10–163.05)	0.365
Weight (kg)	57.00 (53.00–63.68)	56.25 (49.8–62.00)	0.445
**T stage**			
T1	40 (33.6%)	31 (26.1%)	0.561
T2	17 (14.3%)	19 (16.0%)	
T3	4 (3.4%)	5 (4.2%)	
T4	2 (1.7%)	1 (0 .8%)	
**N stage**			
N0	48 (40.3%)	30 (25.2%)	0.064
N1	8 (6.7%)	15 (12.6%)	
N2	3 (2.5%)	8 (6.7%)	
N3	4 (3.4%)	3 (2.5%)	
**M stage**			
M0	63	56	NA
M1	0	0	
**7th AJCC stage**			
I	38 (31.9%)	28 (23.5%)	0.371
II	16 (13.4%)	19 (16.0%)	
III	9 (7.6%)	9 (7.6%)	
**Type of surgery**			
No operation	35 (56.5%)	22 (40.0%)	0.117
Breast conserving surgery	22 (35.5%)	30 (54.5%)	
Mastectomy	5 (8.1%)	3 (5.5%)	
**ER**			
Negative : Positive	2 : 61 (3.2% : 96.8%)	0 : 56 (0% : 100%)	0.186
**PR**			
Negative : Positive	56 : 7 ( 88.9% : 11.1%)	52 : 4 ( 92.9% : 7.1%)	0.458
**HER2/neu**			
Negative : Positive	55 : 8 (87.3% : 12.7%)	43 : 13 (76.8% : 23.2%)	0.135
**Radiotherapy**			
Negative : Positive	11 : 52 (17.5% : 82.5%)	24 : 32 (42.9% : 57.1%)	0.003
**Chemotherapy**			
Negative : Postitive	27 : 36 (42.9% : 51.7%)	24 : 32 (42.9% : 57.1%)	1.00

TMXF, Tamoxifen; GnRH, gonadotrophin releasing hormone; BMI, body mass index; 7th AJCC, to American Joint Committee on Cancer 7th edition; ER, estrogen receptor; PR, progesterone receptor; HER2/neu, Receptor tyrosine-protein kinase erbB-2.

Data reported as number or means ± standard deviation (interquartile range).

### Difference in BMD and TBS between pre-and postmenopausal women

**Figure 1:**
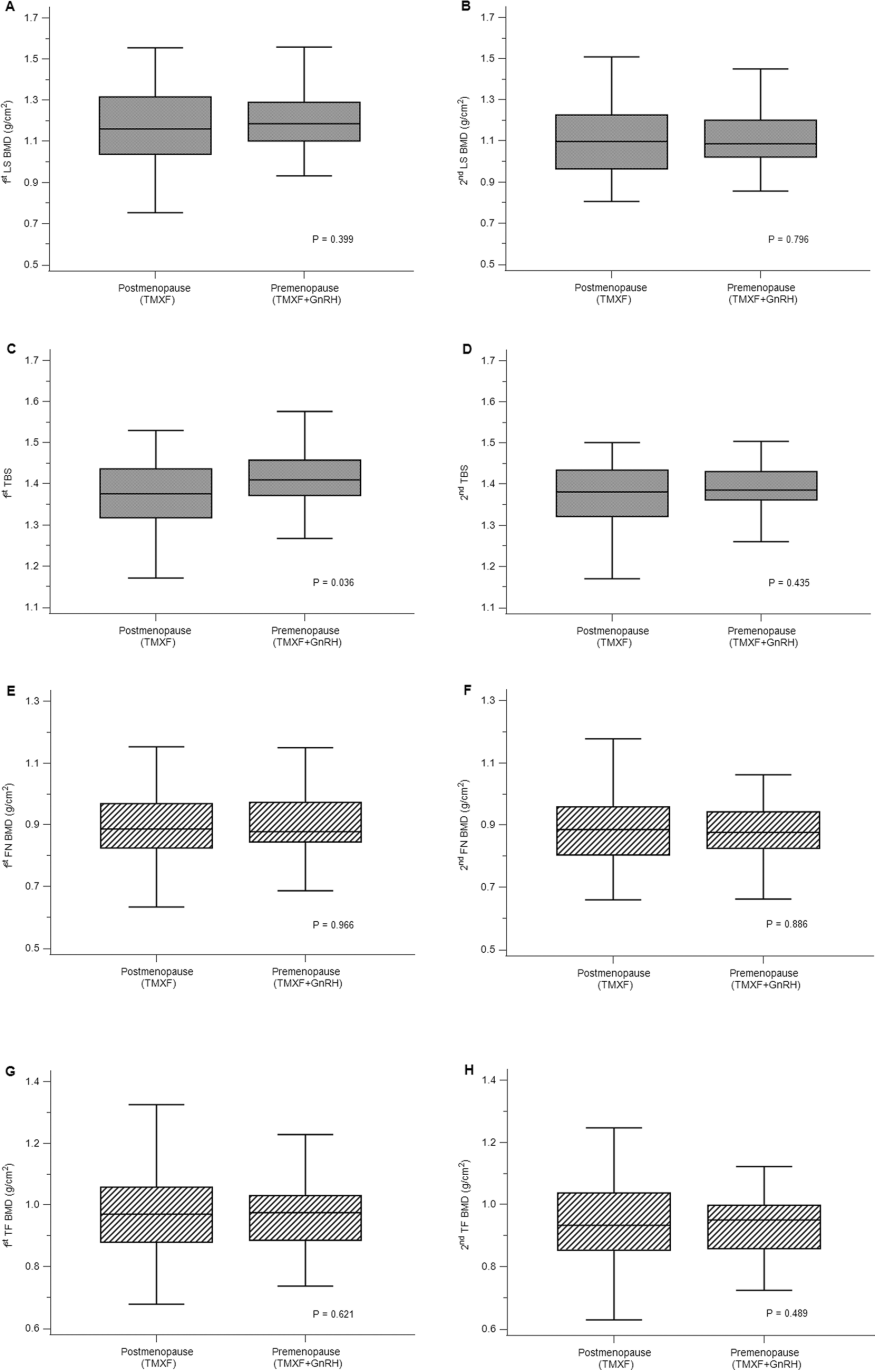
Difference in bone mineral density (BMD, g/cm^2^) and trabecular bone score (TBS) between TMXF group and TMXF + GnRH group in baseline and follow-up dual energy X-ray absorptiometry (DXA). There was no significant difference in BMD values including lumbar spine, femur neck, and total hip between two groups at baseline (**A**,**E**,**G**) and follow-up dual energy X-ray absorptiometry (DXA) (**B**,**F**,**H**). TBS was significantly lower in TMXF group at the baseline study (**C**), however, the difference changed as insignificant in follow-up DXA (**D**).

Prior to breast cancer surgery, baseline BMD values were not significantly different in LS, FN, and TF (median LS, 1.17 [IQR, 1.04–1.31] versus 1.19 [IQR, 1.10–1.29], *P* = 0.399; median FN, 0.89 [IQR, 0.83–0.97] versus 0.88 [IQR, 0.84–0.97], *P* = 0.97; median TF, 0.97 [IQR, 0.88–1.06] versus 0.98 [IQR, 0.89–1.03], *P* = 0.62). In the baseline study, TBS was significantly lower in the TMXF group than in the TMXF + GnRH group (1.37 [IQR, 1.32–1.44] versus 1.41 [IQR, 1.37–1.46], *P* = 0.036). The follow-up DXA results showed similar values of both BMD and TBS comparing the two groups of patients (median LS BMD, 1.10 [IQR, 0.96–1.21] vs. 1.09 [IQR, 1.02–1.20], *P* = 0.796; median FN BMD, 0.89. [IQR, 0.80–0.96] vs. 0.97 [IQR, 0.83–0.93], *P* = 0.89; median TF, 0.93 [IQR, 0.85–1.04] vs. 0.95 [ 0.86–1.00], *P* = 0.49; median TBS, 1.38 [IQR, 1.32–1.43] vs. 1.39 [IQR, 1.36–1.43], *P* = 0.435; Fig. 1 & Table 2).

**Table 2:** Comparative evaluation of DXA related values.

Characteristics		Postmenopause (TMXF)	Premenopause (TMXF + GnRH)	*P* value
No. of patients		63	56	
Follow up period between DXA scans (months)		13.00 (12.00–14.75)	13.50 (12.00–16.00)	0.246
1st DXA scan				
Lumbar spine	BMD values (mg/cm^2^)	1.17 (1.04–1.31)	1.19 (1.10–1.29)	0.398
	Status BMD (n) (Normal BMD: Osteopenia: Osteoporosis:	60 : 1 : 2	55 : 1 : 0	0.404
Femoral neck	BMD values (mg/cm^2^)	0.89 (0.83–0.97)	0.88 (0.84–0.97)	0.966
	Status BMD (n) (Normal BMD: Osteopenia: Osteoporosis:	48 : 14 : 1	48 : 9 : 0	0.429
Total femur	BMD values (mg/cm^2^)	0.97 (0.88–1.06)	0.98 (0.89–1.03)	0.621
	Status BMD (n) (Normal BMD: Osteopenia: Osteoporosis:	53 : 10 : 0	45 : 11 : 0	0.592
TBS	TBS values	1.37 (1.32–1.44)	1.41 (1.37–1.46)	0.036
	Status of TBS (n) (Normal TBS : Degraded TBS : More degraded TBS:	37 : 21 : 5	48 : 8 : 0	0.003
2st DXA scan				
Lumbar spine	BMD values (mg/cm^2^)	1.10 (0.96–1.21)	1.09 (1.02–1.20)	0.686
	Status BMD (n) (Normal BMD: Osteopenia: Osteoporosis:	48 : 12 : 3	51 : 5 : 0	0.061
Femoral neck	BMD values (mg/cm^2^)	0.89 (0.80–0.96)	0.87 (0.83–0.93)	0.886
	Status BMD (n) (Normal BMD: Osteopenia: Osteoporosis:	44 : 18 : 1	43 : 13 : 0	0.335
Total femur	BMD values (mg/cm^2^)	0.93 (0.85–1.04)	0.95 (0.86–1.00)	0.489
	Status BMD (n) (Normal BMD: Osteopenia: Osteoporosis:	51 : 11 : 1	43 : 13 : 0	0.487
TBS	TBS values	1.38 (1.32–1.43)	1.39 (1.36–1.43)	0.436
	Status of TBS (n) (Normal TBS : Degraded TBS : More degraded TBS:	39 : 20 : 4	43 : 12 : 1	0.166

TMXF, Tamoxifen; GnRH, gonadotrophin releasing hormone; BMD, bone mineral density; DXA, dual energy X-ray absorptiometry; TBS, trabecular bone score.

### Changes of BMD and TBS between the 1st and 2nd DXA images

**Figure 2:**
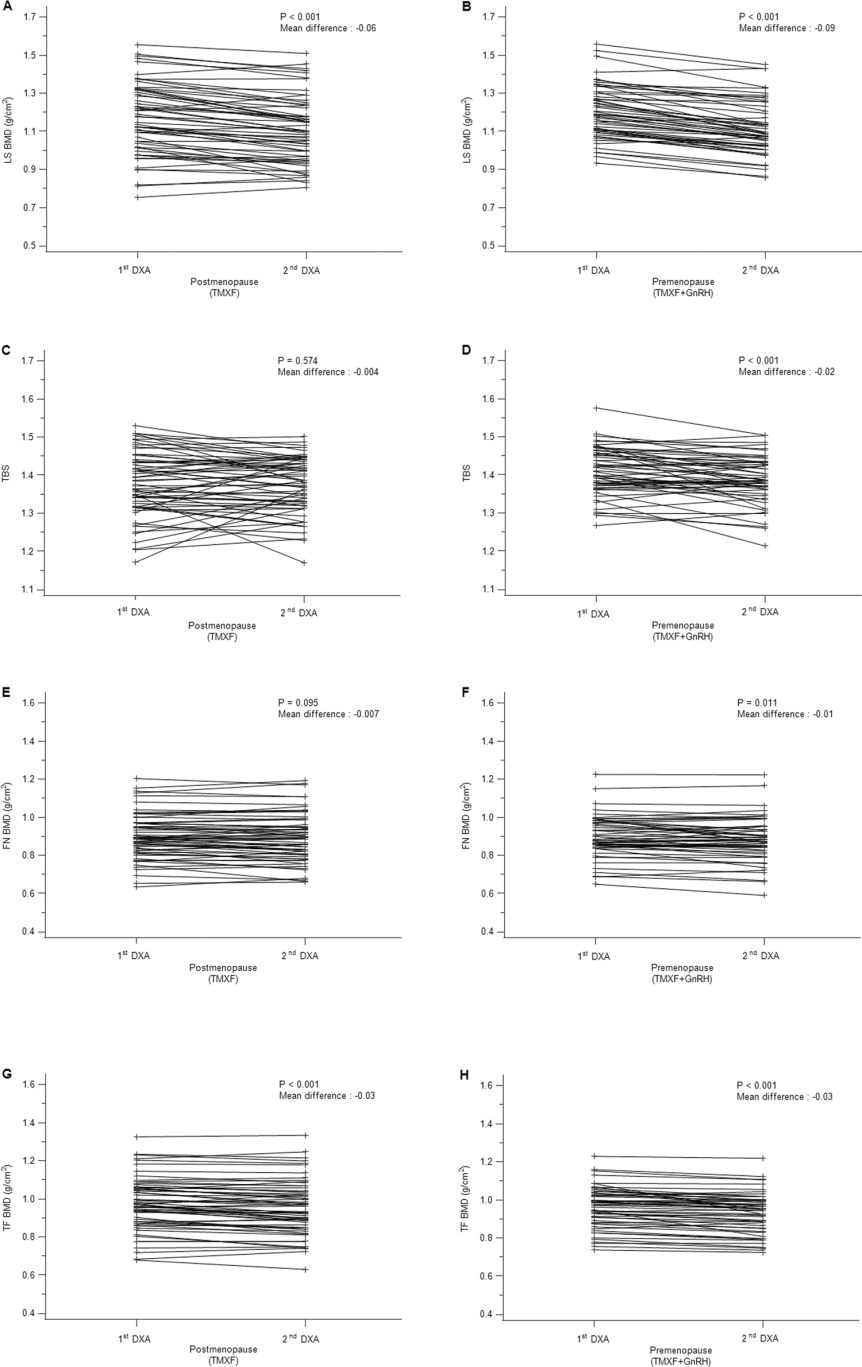
The changes of bone mineral density (BMD, g/cm^2^) and trabecular bone score (TBS) during follow up. Lumbar spine BMD values were significantly decreased in both TMXF group and TMXF + GnRH group (**A**,**B**). TBS did not significantly change in TMXF group (**C**), whereas it was significantly decreased in TMXF + GnRH group (**D**). Femur BMD were significantly decreased during follow up in both TMXF and TMXF + GnRH Group (**F**–**H**, except for femur neck BMD in the TMXF group (**E**).

Comparing the 1st and the 2nd DXA results, LS BMD values were significantly decreased in both the TMXF (*P* < 0.01, mean difference: − 0.06) and TMXF + GnRH groups (*P* < 0.01, mean difference: − 0.09) (Fig. 2A,B). TBS showed no significant change between two scans in the TMXF (*P* = 0.57, mean difference: − 0.004) group, whereas there was significant decrease of TBS in the TMXF + GnRH (*P* < 0.01, mean difference: − 0.02) group during follow-up (Fig. 2C,D). BMD values of femur also showed significant decreasing trend during follow up in both groups, except for FN BMD in the TMXF (*P* = 0.095, mean difference: − 0.007) group. (Fig. 2E–H).

**Figure 3:**
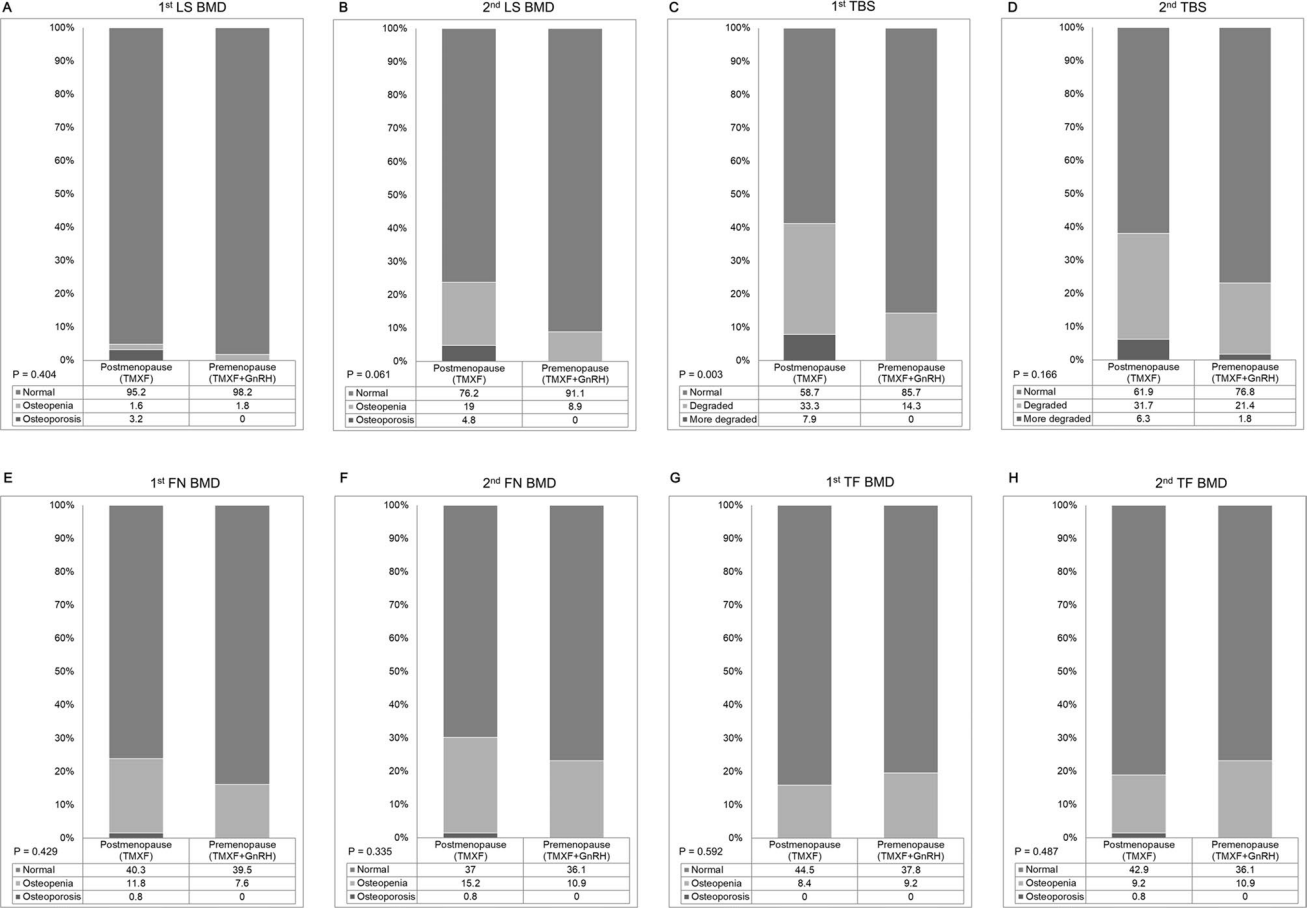
The diagnostic proportion of bone mineral density (BMD, g/cm^2^) and trabecular bone score (TBS) during follow up. The proportion of osteoporosis and osteopenia of lumbar spine BMD was higher in the TMXF group, but not significant (**A**,**B**). Regarding TBS, a significantly higher proportion of the TMXF group are degraded or more degraded status compared with the TMXF + GnRH group at baseline (**C**), but the significance disappeared at the follow-up (**D**). The diagnostic proportion of femur are not difference between two groups at both baseline and follow-up (**E**–**H**).

Using diagnostic criteria of T-scores of LS BMD, the proportion of osteoporosis and osteopenia was higher in the TMXF group; however, there was no statistical significance between the two groups at both baseline (*P* = 0.40) and follow-up (*P* = 0.06) DXA scans. Regarding TBS, a significantly higher proportion of the TMXF patients showed a more degraded state compared with the TMXF + GnRH patients at baseline (37: 21: 5 vs. 48: 8: 0, *P* = 0.003). However, the there was no significant difference at the follow-up DXA scan (*P* = 0.166). The diagnosis of femur did not show difference between two groups at both baseline and follow-up DXA scans. (Table 2 and Fig. 3).

## Discussion

In the present study, BMD was not significantly different between the TXMF group and the TMXF + GnRH group at the baseline DXA, regardless of their age or menopause status. BMD values also showed synchronized decrease, with no distinction between treatment regimens. Although BMD decreased significantly, the amount of the variance of value was within LSC, which could not affect the change of diagnosis of osteoporosis or osteopenia. On the other hand, TBS was significantly different between the two groups at the baseline DXA, but decreased in the TMXF + GnRH group during follow-up. The results imply that GnRH analogs caused more deterioration in bone in terms of bone quality rather than bone mass, after median 14 months of treatment.

Both GnRH analogs and the postmenopausal state may decrease bone mass and degrade microarchitecture because of the estrogen-depriving effect. Estrogen deficiency reduces bone mass, due to its effects of increased bone resorption and increased osteoclastic activity. Also, estrogen deficiency decreases new bone formation due to decreased osteoblastic life^3^. Estrogen inhibits bone resorption by inducing cumulative changes in multiple estrogen-dependent regulatory factors: tumor necrosis factor-α, transforming growth factor-β, and osteoprotegerin (OPG)/receptor activator of nuclear factor kappa-B ligand (RANKL)/RANK system^10^.

The annual BMD percent change in the women who are perimenopausal is greater than that in the women who were postmenopausal^18–21^. A sigmoid pattern of bone loss across menopause has begun 2–3 years before the last menses and ended 3–4 years after the last menses^20^. In the current study, we also found that the decrease of BMD and TBS in LS was greater in the TMXF + GnRH group compared to the TMXF group. In addition, our data shows that the mean change in LS BMD in the TMXF + GnRH group was greater than that of FN and BMD, this is consistent with previous studies that the estrogen deprivation has greater effect on LS bone loss than FN bone loss^19,20^ We presumed that those changes would imply that the deterioration bone status is initiated from the area where the trabecular bone is dominant in young patients with GnRH therapy, which has similar effect on bone status of perimenopausal change.

In premenopausal women, relative rates of bone loss during endocrine therapy were more marked than in postmenopausal women^22^. A previous study reported that TBS is negatively correlated with age^23^. In our study, the baseline TBS of the TMXF group was significantly lower than that of the TMXF + GnRH group before the latter used GnRH analogs; we suspected that this was because of their greater age. However, after use of GnRH analogs, the TBS of the TMXF + GnRH group showed a greater decrease than that of TMXF group. Previous controlled studies also reported that the differences in annual bone loss rate were 1.4% with TMXF, but 5.6% with TMXF and GnRH analog in premenopausal women^22^. In skeletal site-specific response to ovariectomy in a rat model, the trabecular structure shifted from a plate-like to a more vulnerable rod-like shape, which explains the poor bone quality and high fracture risk in patients with ovarian function suppression^11^.

Several clinical studies on breast cancer patients reported that TMXF might have a positive effect on bone metabolism^2,24–26^. In a prospective study with 44 postmenopausal women with hormone-receptor positive breast cancer, BMD was minimally increased in the LS and FN after 12 months of treatment with TMXF, with a significant decrease in osteocalcin levels^2^. Postmenopausal women showed that TMXF increases areal BMD and reduces fracture risk compared to placebo (relative risk 0.68, 95% confidence interval 0.51–0.92)^16^. Experimental studies on ovariectomized rat models also reported that TMXF had a protective effect for bone^3,27–29^. After ovariectomy, TMXF administration in rats induced collagen fibers to increase in the periphery of the compact bone, osteoblastic activity increased, matrix released, osteocyte differentiated, and osteoclast cells decreased^3^.

As osteoporosis is characterized by both loss of bone mass and architectural deterioration, BMD alone has a limitation for predicting fracture risk. For the evaluation of bone microarchitecture of LS, TBS, a novel gray-level texture measurement, was developed to be extracted from DXA images^30^. Higher scores of TBS correlate with fracture-resistant microarchitectures, whereas lower scores reflect fracture-susceptible microarchitecture, despite identical BMD^31^. Previous studies reported inconsistent results about BMD and TBS. Our previous study reported that after thyroid-stimulating hormone therapy, although the average areal BMD of patients was not significantly changed, the mean TBS significantly decreased^32^. The other study reported that after bisphosphonate treatment, BMD decreased and TBS increased during a 3-year follow up period^33^.

Previous studies also reported the sensitivity of TBS in predicting fracture risk in other endocrine diseases. The prevalence of vertebral fracture is higher in patients with primary aldosteronism^34,35^. Plasma aldosterone concentrations were inversely correlated with the TBS (*P* = 0.028) but not with bone mass in women^36^. TBS is more sensitive for predicting osteoporotic fracture than BMD in diabetes^37^. For patients with breast cancer, consistently, our study showed increased TBS in some TMXF group as the positive effect of TMXF.

Our study had several strengths. We investigated and compared both bone quality and bone density in the premenopausal women who received combination treatment with TMXF and GnRH analogs and the postmenopausal women treated with only TMXF. The numbers of enrolled subjects were relatively larger than in previous studies^16,38^.

Our study also had some limitations. Because of its retrospective design, bone turnover markers were excluded because they were not routinely measured in our practical clinical setting. The follow-up period for our study was also relatively short. However, it is known that bone loss is most marked in the first year of endocrine therapy for breast cancer and is particularly evident at the LS^39^. Finally, there were no data available on fragility fractures during follow-up because no clinical data regarding fracture history were available. More prospective studies of TBS and the correlation with the risk of fracture are needed to confirm our findings.

The present study found that TMXF had a positive effect on bone microarchitecture for postmenopausal breast cancer patients. On the other hand, estrogen deprivation by GnRH analogs reduced bone microarchitecture in premenopausal breast cancer patients, even those who were receiving combination therapy with TMXF. The results of our study showed that TBS had changed more sensitively compared with BMD. Therefore, it may be helpful to consider the possibility of using TBS as a more sensitive measure of bone microarchitecture, especially for premenopausal patients receiving hormone therapy with GnRH analogs.
