# Author Correction: Effect of tamoxifen with or without gonadotropin-releasing hormone analog on DXA values in women with breast cancer

**DOI:** 10.1038/s41598-023-38676-8

**Published:** 2023-08-07

**Authors:** Eun Heui Kim, Yun Kyung Jeon, Kyoungjune Pak, Taewoo Kang, Kyung-Eun Kim, Seong-Jang Kim, In-Joo Kim, Keunyoung Kim

**Affiliations:** 1 Division of Endocrinology and Metabolism, Department of Internal Medicine and Biomedical Research Institute, Pusan National University Hospital, Busan, Republic of Korea; 2 Department of Nuclear Medicine and Biomedical Research Institute, Pusan National University Hospital and School of Medicine, Pusan National University, Busan, Republic of Korea; 3 Busan Cancer Center (Breast Cancer Clinic) and Biomedical Research Institute, Pusan National University Hospital, Busan, Republic of Korea; 4 Department of Nuclear Medicine and Research Institute for Convergence of Biomedical Science and Technology, Yangsan Pusan National University Hospital, Yangsan, Republic of Korea

Correction to: *Scientific Reports* 10.1038/s41598-021-82824-x, published online 09 February 2021

In the original version of this Article, Affiliation 2 was incorrectly given as ‘Department of Nuclear Medicine and Biomedical Research Institute, Pusan National University Hospital, Busan, Republic of Korea’. The correct affiliation is:

Department of Nuclear Medicine and Biomedical Research Institute, Pusan National University Hospital and School of Medicine, Pusan National University, Busan, Republic of Korea

The original Article has been corrected.

